# Metabolite Profiling to Characterize Disease-related Bacteria: GLUCONATE EXCRETION BY PSEUDOMONAS AERUGINOSA MUTANTS AND CLINICAL ISOLATES FROM CYSTIC FIBROSIS PATIENTS*[Fn FN2]

**DOI:** 10.1074/jbc.M112.442814

**Published:** 2013-04-09

**Authors:** Volker Behrends, Thomas J. Bell, Manuel Liebeke, Anne Cordes-Blauert, Syedah N. Ashraf, Chandrika Nair, James E. A. Zlosnik, Huw D. Williams, Jacob G. Bundy

**Affiliations:** From the ‡ Department of Surgery and Cancer and; § Department of Life Sciences, Imperial College London, London SW7 2AZ, United Kingdom and; the ¶ Center for Understanding and Preventing Infection in Children, Department of Pediatrics, Faculty of Medicine, University of British Columbia, Vancouver V5Z 4H4, Canada

**Keywords:** Functional Genomics, Gene Regulation, Metabolism, Metabolomics, Pseudomonas aeruginosa, Metabolic Footprinting

## Abstract

Metabolic footprinting of supernatants has been proposed as a tool for assigning gene function. We used NMR spectroscopy to measure the exometabolome of 86 single-gene transposon insertion mutant strains (mutants from central carbon metabolism and regulatory mutants) of the opportunistic pathogen *Pseudomonas aeruginosa*, grown on a medium designed to represent the nutritional content of cystic fibrosis sputum. Functionally related genes had similar metabolic profiles. *E.g.* for two-component system mutants, the cognate response regulator and sensor kinase genes clustered tightly together. Some strains had metabolic phenotypes (metabotypes) that could be related to the known gene function. *E.g.* pyruvate dehydrogenase mutants accumulated large amounts of pyruvate in the medium. In other cases, the metabolic phenotypes were not easily interpretable. The *rpoN* mutant, which lacks the alternative σ factor RpoN (σ^54^), accumulated high levels of gluconate in the medium. In addition, endometabolome profiling of intracellular metabolites identified a number of systemic metabolic changes. We linked this to indirect regulation of the catabolite repression protein Crc via the non-coding RNA *crcZ* and found that a *crcZ* (but not *crc*) mutant also shared the high-gluconate phenotype. We profiled an additional set of relevant metabolic enzymes and transporters, including Crc targets, and showed that the Crc-regulated *edd* mutant (gluconate-6-phosphate dehydratase) had similar gluconate levels as the *rpoN* mutant. Finally, a set of clinical isolates showed patient- and random amplification of polymorphic DNA (RAPD) type-specific differences in gluconate production, which were associated significantly with resistance across four antibiotics (tobramycin, ciprofloxacin, aztreonam, and imipenem), indicating that this has potential clinical relevance.

## Teaser

**Background:** Phenotypic profiling of knockout libraries is a possible functional genomics strategy.

**Results:** Gluconate excretion is a novel phenotype of the *Pseudomonas aeruginosa rpoN* mutant, which is also weakly associated with antibiotic susceptibility in a clinical strain panel.

**Conclusion:** The *rpoN* phenotype results from catabolite repression deregulation of 6-phosphogluconate dehydratase.

**Significance:** NMR profiling can uncover novel gene functions with potential clinical relevance.

## Introduction

*Pseudomonas aeruginosa* is an ecologically versatile, Gram-negative bacterium, and some of this versatility is undoubtedly due to its ability to use a wide range of different compounds as energy sources for growth. It does not normally cause disease in healthy human adults, but can infect cuts and burns. It also frequently forms chronic infections in the lungs of cystic fibrosis (CF)^3^ patients. CF is a genetic disease caused by mutation of the cystic fibrosis transmembrane conductance regulator, a chloride ion transporter, that leads to an ionic imbalance across the apical surface of epithelial cells and a dehydrated airway surface that impairs mucociliatory clearance. This offers an opportunity for bacterial colonization, and CF patients typically have a wide range of different colonizing bacteria (1). However, the majority of CF patients eventually become infected with *P. aeruginosa*, which is very difficult to eradicate from the CF lung once established and typically forms life-long chronic infections. The majority of CF patients ultimately die from respiratory failure caused by the cycle of infection, inflammation, and tissue damage brought on by this (2). Understanding how *P. aeruginosa* adapts to the changing nutritional and physical environment of the CF lung as well as the presence of competing microbes is important to understand the ecology of this infection. Some genetic adaptations of *P. aeruginosa* have been identified during its chronic infection of the CF lung, but further knowledge of these and other aspects of the biology of this versatile bacterium will improve understanding of its infection biology as well as basic aspects of its gene function and physiology.

The compendium approach has been influential in the post-genomic era. The basic concept is that compendia of phenotypic profiles, such as those generated by transcriptional microarrays, can be used to classify single-gene knockout strains on the basis that functionally related genes would cluster together (3). This approach is not restricted to transcriptomic data. The same approach can be applied to metabolomic profiles. Indeed, it has been argued that there are advantages to using metabolome rather than transcriptome data, as metabolic control analysis predicts that single-gene knockouts are likely to have larger changes in metabolite than transcript levels (4). The experimental evidence to date is compatible with this, and the metabolic effects of gene deletion are clearly greater than the effects of altering growth rate (5). There are also advantages to analyzing the exometabolome of cells, or metabolic footprinting (6). These are both procedural (the separation of medium from the cells is straightforward, and the accumulated metabolic changes are effectively integrated over time) and biological (as the extracellular medium is not under cellular homeostatic control, metabolites can accumulate to high levels). *P. aeruginosa* has a whole-genome knockout library available (PA14NR) (7). Previous functional genomic studies with this library have focused on simple phenotypic endpoints (such as growth) under multiple conditions (8–10). These approaches to characterizing “phenotypic landscapes” can be very powerful (11, 12), but it would also be valuable to compare the compendium approach on the basis of information-rich complex phenotypes.

Here we investigated the feasibility of using a metabolic footprinting approach to characterize large sample sets like whole-genome knockout libraries. As a proof-of-principle study, we carried out metabolic footprinting by NMR spectroscopy on a subset of 86 mutant strains of *P. aeruginosa*, chosen to represent a range of targets, including metabolic enzymes, transcriptional regulators, two-component systems, and quorum sensing pathways. We then analyzed the exometabolome of cells grown on synthetic cystic fibrosis medium (SCFM), a defined medium that is designed to mimic the metabolic composition of CF sputum (13). Finally, we tested a novel phenotype (gluconate excretion) in a number of CF clinical isolates and its relation to antibiotic susceptibility.

## EXPERIMENTAL PROCEDURES

### 

#### 

##### Strains and Growth Conditions

The deletion library strains were taken from PA14NR, created by Liberati *et al.* (7), in a PA14 background. We chose 86 of these strains. The full list is given in supplemental Table S1. For the characterization of the *rpoN* mutation and the *crc* and *crcZ* genes, we received an in-frame deletion mutant and complemented mutant strain in the PAO1 background from D. Haas (University of Lausanne, Switzerland). To account for the functional glutamine auxotrophy (14), we supplemented the growth media for all experiments containing the *rpoN* mutant with 2 mm glutamine. All strains were grown in 1 ml of SCFM (13) in 96-well deep-well plates sealed with oxygen-permeable membranes to allow gas transfer. Samples were taken after 24 h by centrifuging the plates and transferring the supernatants to a new deep-well plate. Experiments were carried out with five biological replicates.

##### NMR Spectroscopy

We prepared samples for NMR by mixing 0.8 ml of cell supernatant with 0.2 ml of NMR buffer. The NMR buffer contained 3.3 mm sodium trimethylsilyl-^2^H_6_-propanesulfonic acid and 24 mm NaN_3_ in ^2^H_2_O, resulting in a final concentration in each sample of 0.66 and 4.8 mm, respectively. We acquired the spectra on a Bruker DRX600 Avance spectrometer (Bruker Biospin, Rheinstetten, Germany) with a magnetic field strength of 14.1 tesla and 600 MHz proton resonance frequency, equipped with a 4-mm flow probe. The samples were held at 300 K during acquisition and were introduced using a Gilson robot flow-injection autosampler. A sample volume of 0.7 ml was injected onto the flow probe, with three probe wash steps between every sample. The samples were injected in the order that they were cultured in 96-well plates, and five replicate plates were injected in turn. Spectra were acquired using a one-dimensional solvent-suppressed sequence essentially as described by Beckonert *et al.* (15).

Metabolite assignments were on the basis of matching chemical shifts and multiplicities to online databases such as the Human Metabolome Database (16) and the Biological Magnetic Resonance Bank (17) and by using the proprietary NMR Suite database (Chenomx, Alberta, Canada). Assignment of pyruvate, 2,2′-dihydropropanoate and gluconate was confirmed by acquiring two-dimensional heteronuclear single quantum coherence spectra.

##### NMR Data Processing

The data were initially processed in iNMR 3 (Nucleomatica, Molfetta, Italy). The free induction decays were zero-filled by a factor of 1.5 and multiplied by an exponential apodization factor equivalent to 1 Hz line broadening, followed by Fourier transform. Phase correction, baseline correction, and referencing chemical shifts to sodium trimethylsilyl-^2^H_6_-propanesulfonic acid (δ = 0) were carried out using the proprietary algorithms of the software, and the spectra were exported as ASCII files and imported into Matlab. For first-pass data analysis, the spectra were then aligned following the method of Veselkov *et al.* (18), and integral boundaries were chosen manually so that, as far as possible, each resonance was represented by a single integral, and all resonances across the set of spectra were included. In many cases, integrals included contributions from more than one metabolite because of overlapping resonances. The spectra were then reprocessed for a more in-depth analysis by fitting individual metabolites to the spectra using the R package BATMAN, which uses a Bayesian approach to deconvolve one-dimensional spectra into a mixture of Lorentzian functions with wavelet functions used to fit residual intensity (19, 20). The spectra were ordered with respect to pH to improve peak identification (21).

##### Gluconate Assay

We used a commercial enzymatic gluconate assay (Megazyme Ltd., Ireland). The assay was carried out according to the instructions of the manufacturer, except that we converted it to a 96-well-plate format by reducing the reaction mixture volume to 20 μl of reaction buffer, 20 μl of NADP^+^/ATP solution, 2 μl of 6-phosphogluconate dehydrogenase suspension, 2 μl of gluconate kinase suspension, and 100 μl of bacterial supernatant. The assay was linear between 5 and 1250 μm gluconate.

##### Oxygen Consumption Assay

We grew 50-ml cultures of the PA14 wild-type strain and the *rpoN* mutant and harvested them by centrifugation (3000 × *g*, 30 min) when they reached an *A*_600_ of between 0.3 and 0.8. The pelleted cells were washed in 20 ml of phosphate buffer (pH 7), resuspended in 500 μl of phosphate buffer, and kept on ice. An oxygen electrode was equilibrated with 3 ml of phosphate buffer (pH 7) and maintained at 30 °C. Briefly, 100 μl of cells was added to 3 ml of phosphate buffer on the electrode, and the signal response was recorded on a chart recorder for 3 min to establish a base-line oxygen concentration. For each sample, 30 μl of either 1 m glucose or 1 m gluconate was then added, and the signal response was recorded for 3 min to establish the rate of oxygen consumption associated with glucose or gluconate dehydrogenase, respectively. The base-line rate of oxygen consumption was subtracted from the rate following addition of either glucose or gluconate. Oxygen consumption rates were then normalized to protein concentration for each sample, which was determined using a modified Lowry procedure (22).

##### Substrate Utilization Profiling

We used the commercial Biolog “phenotype microarray” system (23) to profile the wild-type and *rpoN* mutant strains (on a PA14 background) with the plates PM1, PM2A, and PM3B containing 190 separate sole carbon source and 95 separate sole nitrogen source substrates (Technopath, Ballina, Ireland). Briefly, cells were grown as preculture in M9 + glucose + glutamine to optical densities of 0.6–0.8. At this optical density, 2 ml of cells were harvested, washed with quarter-strength microbiological Ringer's solution, and resuspended in 10 ml of inoculation medium (M9 salts). For carbon plates, the inoculation medium contained NH_4_Cl and glutamine, and for PM3B, it contained glucose and glutamine. Cells were grown for 24 h, after which the optical density at 595 and 750 nm was recorded. The 750-nm reading is a measurement of general turbidity, whereas 595 nm is the absorption maximum of the reduced dye.

##### Metabolite Profiling Using GC-MS

We analyzed the endometabolome of the wild-type, *rpoN*, and complemented mutant on the PAO1 background. The cells were sampled (*n* = 6) by rapid filtration using a method adapted from Bolten *et al.* (24). Briefly, 2.5 ml of culture was harvested by vacuum filtration (filter: polytetrafluoroethylene, 0.45-μm pore size, 47 mm; stand: magnetic filter funnel, Pall, Ann Arbor, MI) and washed with 5 ml of 0.9% saline solution. The filter was transferred to a 50-ml reaction tube containing 10 ml of cold (−40 °C) methanol:acetonitrile:H_2_O (2:2:1, v/v/v) and frozen in liquid nitrogen. Afterward, all extracts were subjected to two freeze-thaw cycles and sonication. After the removal of the filter, the extracts were centrifuged to pellet the cellular debris and dried in a vacuum concentrator (Eppendorf, 45 °C). We derivatized the samples by methoximation followed by trimethylsilylation, following the method of Kind *et al.* (25), and analyzed them on a 7890 GC coupled to a 5975c mass spectrometer (Agilent, UK). We analyzed the data by first processing all files with AMDIS using the FiehnLib retention time-locked library (25). We then carried out an additional step for manual inspection of all peaks across all samples, with manual adjustment of retention time windows where appropriate, and “back-filled” the sample matrix by reintegrating the raw data so that every sample/peak combination had a numerical value (26).

##### Measuring Antibiotic Susceptibility

We measured gluconate production in 156 longitudinal isolates taken from 16 patients, in which each set of strains from a single patient represented a clonal lineage (27). We then chose a subset of 96 of these isolates and measured minimal inhibitory concentrations of five different antibiotics (tobramycin, imipenem, ciprofloxacin, colistin, and aztreonam) using Etest strips impregnated with an antibiotic gradient (bioMérieux, Basingstoke, UK). Genotypes as assessed by random amplification of polymorphic DNA (RAPD) typing (28) of these isolates were available from a previous study (29).

## RESULTS

### 

#### 

##### Growth and Sampling

We selected a total of 86 mutants, corresponding to 72 genes in total, as some genes were represented by more than one insertion (supplemental Table S1). We grew the bacterial strains in 1 ml of SCFM and sampled the medium after 24 h of growth for exometabolome profiling.

Most of the strains alkalized the growth medium to some extent (data not shown), and so there were pH-related shifts in resonance frequencies between spectra. Because of this, using simple peak integrals gave poor-quality data, and so we used a peak-fitting deconvolution method to provide the best output. Manual peak-fitting (“targeted profiling”) by a skilled spectroscopist using computer-aided software such as Chenomx NMR Suite (30) is highly precise (31), but is time-consuming and therefore not feasible for projects with large numbers of spectra. We used the freely available R package BATMAN (19, 20) to fit individual metabolites to the spectra, thus allowing for both peak overlap and peak shifting. We manually inspected all of the spectra to make sure there were no incorrectly fitted metabolites. The results were quantitatively comparable with the *de facto* gold standard Chenomx data when compared for a representative sample of spectra (data not shown). We quantified 25 metabolites from the spectra, eight of which were excreted by some or all of the strains and 17 of which were present in the original medium and consumed to varying degrees. All of the data are available for downloading (supplemental Table S2). Five of the eight excreted metabolites were assigned (ethanol, formate, acetate, pyruvate, and gluconate), and we also quantified three unassigned resonances (δ 1.06 ppm (doublet), δ 2.47 ppm (singlet), and δ 4.44 ppm (singlet)).

##### Biological Validation by Functional Clustering

**FIGURE 1.**
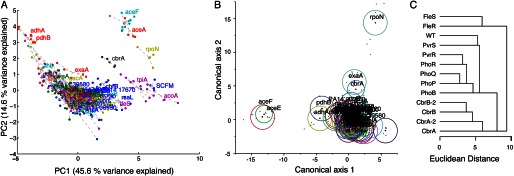
**Functionally related *P. aeruginosa* strains are metabolically similar.** *A*, principal component analysis score plot of all strains, PCs 1 and 2. Different colors represent different mutant strains, and the lines connect individual points to strain centroids. *B*, linear discriminant analysis of dimension-reduced data (PCs 2–10 inclusive). *Ellipses* represent 95% confidence intervals for strains. *C*, hierarchical cluster analysis of mean concentrations for two-component system mutants.

**FIGURE 2.**
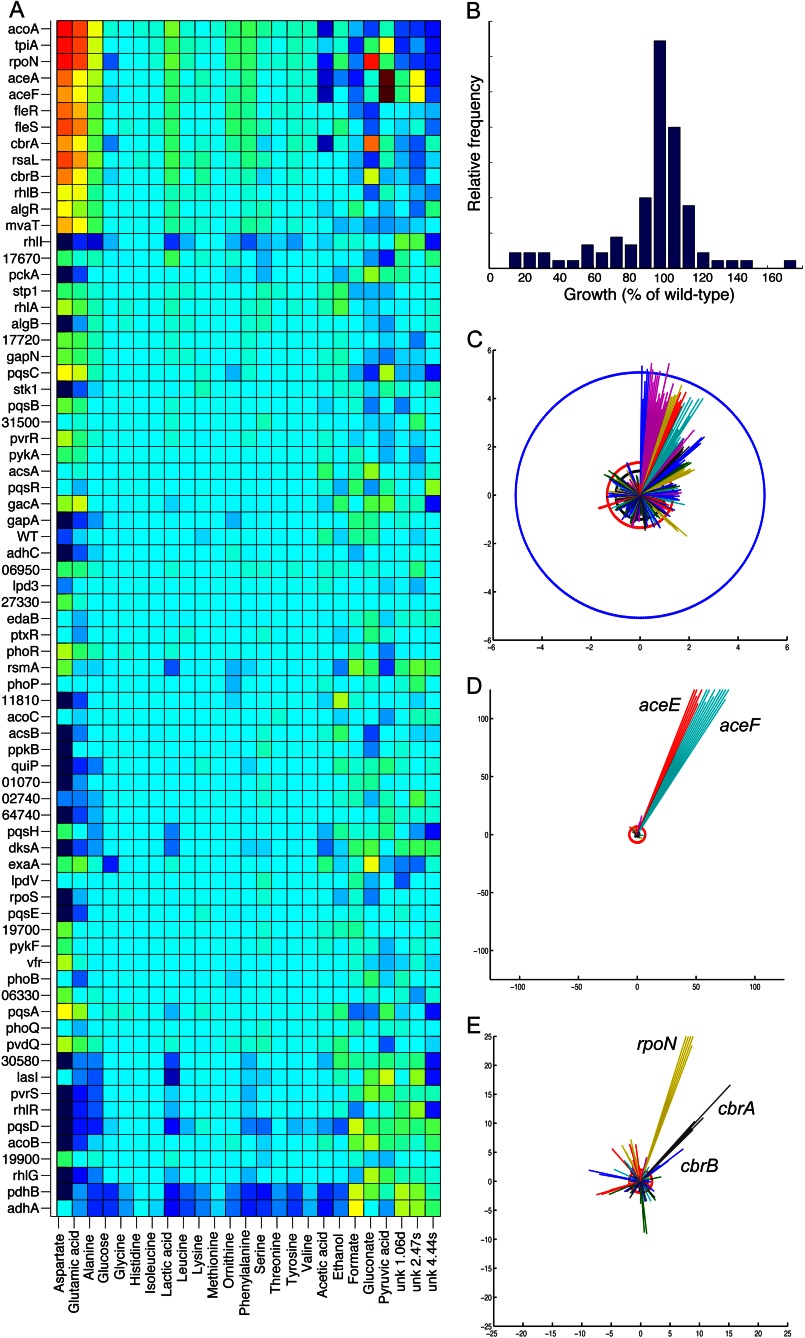
**Metabolite changes in *P. aeruginosa* mutants.** *A*, metabolite levels for all strains (data normalized to medians). *B*, growth of all mutants (as percent of the wild type). *C*, alanine utilization increases with extent of growth. In the “sunburst” plot, each ray represents a single sample. Different mutant strains are indicated by different colors and are sorted clockwise according to increasing *A*_600_. The *blue circle* indicates the original level in SCFM, the *red circle* represents the mean final level, and the *black circle* the median final level across all strains. *D*, *aceE* and *aceF* strains have high levels of pyruvate excretion. *E*, the *rpoN* strain produces high levels of gluconate and the *cbrA* strain the next highest.

We wanted to determine whether NMR exometabolome data would be useful for clustering knockout mutants of related function, and so carried out an initial analysis on whole profiles. Principal component analysis is a robust, unsupervised, multivariate technique that is often used for dimension reduction and data visualization. Principal component analysis alone was sufficient to show that strain replicates clustered tightly together, indicating good biological reproducibility (Fig. 1). The majority of the mutants grew to similar final *A*_600_ as the wild type (Fig. 2), but some strains had clear growth defects. The two *tpiA* mutants and putative *acoA* mutant did not grow, whereas the *rpoN*, *aceE*, and *aceF* mutants had moderate to severe growth defects. A number of strains grew to higher final densities than the wild type, with six mutants reaching *A*_600_ of greater than 120% of the wild type. PC1 could be interpreted as an overall growth axis. PC1 scores were highly correlated to *A*_600_ (R^2^ = 0.82, *p* < 0.0001). On PC2, the *aceE*, *aceF*, as well as *rpoN* clustered away from the majority of the strains (Fig. 1).

We followed the approach of Raamsdonk *et al.* (4) in using linear discriminant analysis, which is supervised in the sense that it attempts to separate all strains in the biochemical space but unsupervised with respect to any knowledge of which strains are similar. Hence, if two strains cluster together following linear discriminant analysis, it can be interpreted as an unsupervised indication that the strains have a similar metabolism. Furthermore, this could be used to disentangle strain-specific changes from the overall effects of growth. Principal component analysis can be used as a dimension reduction preprocessing step for linear discriminant analysis, and excluding PC1 effectively removes the growth-related information. Using all the data as an input, the *aceE* and *aceF* strains were separated from the rest along axis 1, and *rpoN* was separated from the rest along axis 2 (data not shown). This remained true even when using the first 10 PC scores as input but excluding PC1 (Fig. 1).

In addition, we focused on two-component systems. These bacterial regulatory systems prototypically comprise a membrane-localized histidine sensor kinase and a cytoplasmically localized response regulator (32). They have low levels of cross-talk (33), so they can be used for biological validation (*i.e.* do kinase and regulator pairs cluster together?). We clustered the two-component system mutants separately on the basis of Euclidean distance. The kinase/regulator pairs formed separate clusters when there were significant differences from the wild type, *i.e.* for the *cbrA*/*cbrB* and *fleR*/*fleS* pairs (Fig. 1). Presumably the other genes were not expressed under these growth conditions. Together, all of these observations provide a biological validation that functionally related strains clustered together.

##### Metabolic Effects of Mutations

We then examined the data in more depth to get a more specific idea of which metabolites were altered and for which strains (Fig. 2). Alanine, lactate, phenylalanine, and glutamate were strongly negatively correlated with growth. *I.e.* lower levels in the supernatant (and therefore elevated compound uptake) coincided with higher optical densities. This is obvious by eye when examining the data sorted by *A*_600_ (*e.g.* for alanine, Fig. 2*C*). Lactate is the major carbon source in SCFM, and lactate utilization and growth were strongly correlated across most strains. Some strains, however, most notably strains with mutations in genes encoding for quorum sensing molecule synthesis (*rhlI*, *lasI*, *pqsA*, and *pqsH*), had inefficient lactate use. These strains consumed almost all the lactate from the medium but did not grow to the OD_600_ that would have been expected (data not shown). The only other strains with equally inefficient lactate utilization were mutants deficient in RsmA and DksA, both negative regulators of quorum sensing (34–36).

**FIGURE 3.**
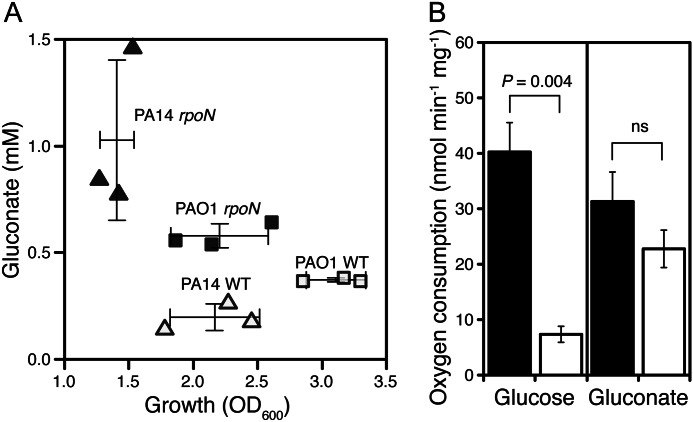
**RpoN deletion decreases growth but increases gluconate excretion in two different strain background (*A*).** ▴ and ■, *rpoN* mutant strains; ▵ and □, wild-type strains; ▴ and ▵, PA14 background; ■ and □, PAO1 background. *Bars* show mean ± S.E., *n* = 3. *B*, glucose dehydrogenase but not gluconate dehydrogenase activity is deregulated in *rpoN*-mutant strains. *Bars* show mean ± S.E., *n* = 3. *Filled bars*, *rpoN* mutant; *empty bars*, wild type; *ns*, not significant.

Two of the excreted metabolites were present at very high levels in some strains: gluconate and pyruvate. Pyruvate was excreted by the *aceE* and *aceF* mutants, respectively, whereas gluconate was mainly produced by the *rpoN*, *cbrA*, and *cbrB* mutants and was highest in the *rpoN* mutant in particular (Fig. 2). We validated this observation independently by carrying out an enzymatic gluconate quantification of all samples. This confirmed the NMR results, with the *rpoN* strain samples again with the highest gluconate production (data not shown). Gluconate production has not been described as an RpoN-dependent phenotype that we are aware of, and so we wanted to confirm that this was a genuine effect of the *rpoN* mutation and not a downstream effect of the transposon insertion or secondary mutations. We measured growth and gluconate for samples grown in standard shake flasks, both for the library *rpoN* transposon mutant and the PA14 wild type as well as for a clean in-frame *rpoN* deletion mutant in the PAO1 background and its isogenic parent strain. We supplemented the medium with 2 mm glutamine to make sure that any phenotype was not a simple consequence of glutamine auxotrophy (37). Growth was decreased, and gluconate was elevated in both backgrounds (Fig. 3), and so we conclude that gluconate production is a reproducible RpoN-defective phenotype.

##### Characterization of the rpoN Mutation

To further characterize the pleiotropic effects of the *rpoN* mutation, we analyzed the metabolic capacity of the *rpoN* mutant and its parent strain using the phenotypic microarray system from Biolog (Technopath). This technology measures microbial activity using a redox-active dye in a 96-well format, with each plate containing 95 different conditions (23). We investigated activity on 190 different sole carbon sources (using glutamine as a nitrogen source) and 95 different sole nitrogen sources (using glucose as a carbon source). This confirmed the key role of *rpoN* in nitrogen metabolism, with the mutant only able to utilize 23 nitrogen sources, whereas the wild type utilized 52. Surprisingly, the *rpoN* mutant exhibited a higher activity than the wild type on many of the carbon sources, with detectable activity on 117 and 75 carbon sources, respectively (supplemental Table S3).

**FIGURE 4.**
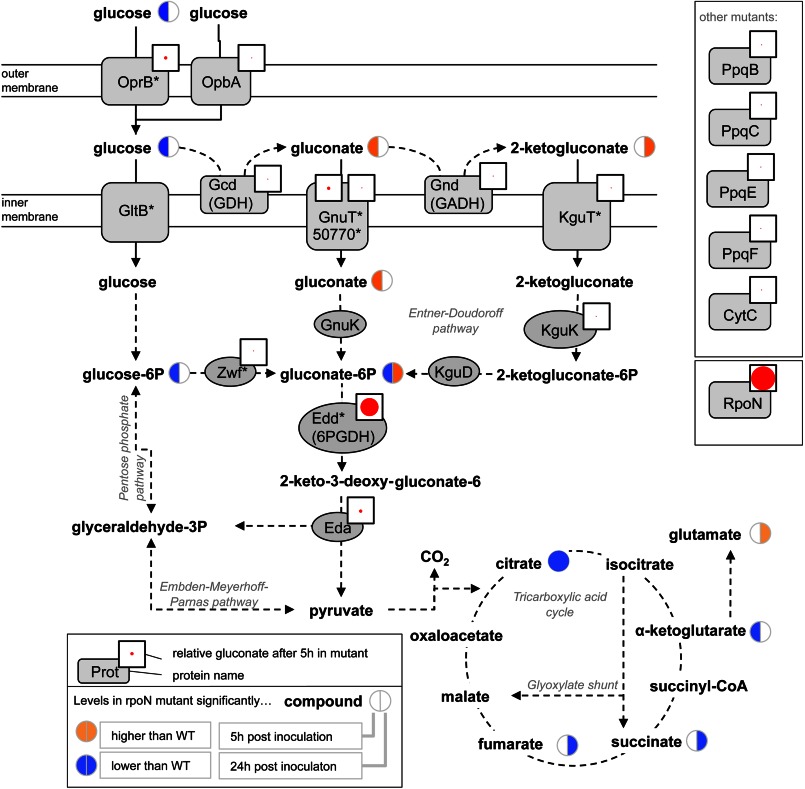
**The 6-phosphogluconate dehydratase (*edd* mutant) is the only mutant with a Crc-binding motif tested that gives the same gluconate accumulation phenotype as the *rpoN* mutant.** Extracellular gluconate concentrations for mutant strains are given by the size of the *red dot* in a *white square*. Additionally, exo- and endometabolome changes for the *rpoN* mutant are shown simultaneously in the same pathway context. Key given in *inset*. Gene names are taken from the *Pseudomonas* genome database. An *asterisk* after the name indicates a predicted Crc-binding motif, taken from Browne *et al.* (56).

We also looked at the intracellular metabolome (endometabolome) of the *rpoN* clean deletion mutant, reanalyzed the supernatants (exometabolome) in more detail, and compared the profiles to the wild type and a complemented mutant strain. To this end, we grew the strains in SCFM in shake flasks, sampled them at 5 h and 24 h by rapid filtration, and analyzed the endometabolome by GC-MS and the exometabolome by NMR. The metabolic effects of the *rpoN* mutation were both wide-ranging and growth phase-dependent (Fig. 4). The exometabolome data showed that organic acid production is altered in the *rpoN* mutant, which produced more gluconate but less acetate levels than the WT after 5 h. In addition, proline and serine utilization was reduced beyond what would be expected for the lower growth rate. In contrast, glucose utilization was increased. After 24 h, most metabolites were used up, but the *rpoN* mutant failed to utilize all of the available histidine (data not shown).

After 5 h of growth, the main endometabolome phenotypes included deregulation of central sugar metabolism (higher levels of gluconate and lower levels of glucose-6-phosphate) as well as citric acid cycle metabolites (lower levels of citrate and α-ketoglutarate). After 24 h of growth, more significant differences could be observed, with amino acid metabolism and the citric acid cycle particularly affected (Fig. 4). The *rpoN* mutant also contained significantly lower levels of disaccharides such as trehalose and sucrose. However, few metabolites showed consistent differences across both time-points. Only citrate and 4-hydroxyphenylacetate had consistently lower levels (data not shown). The effect on 4-hydroxyphenylacetate suggests a possible role for RpoN in regulating the conversion of phenylalanine and tyrosine. This is further strengthened by the fact that the tyrosine/phenylalanine ratio was about 10 times higher in the *rpoN* mutant than in the wild type and complemented strains after 24 h of growth (data not shown).

##### Gluconate Production

**FIGURE 5.**
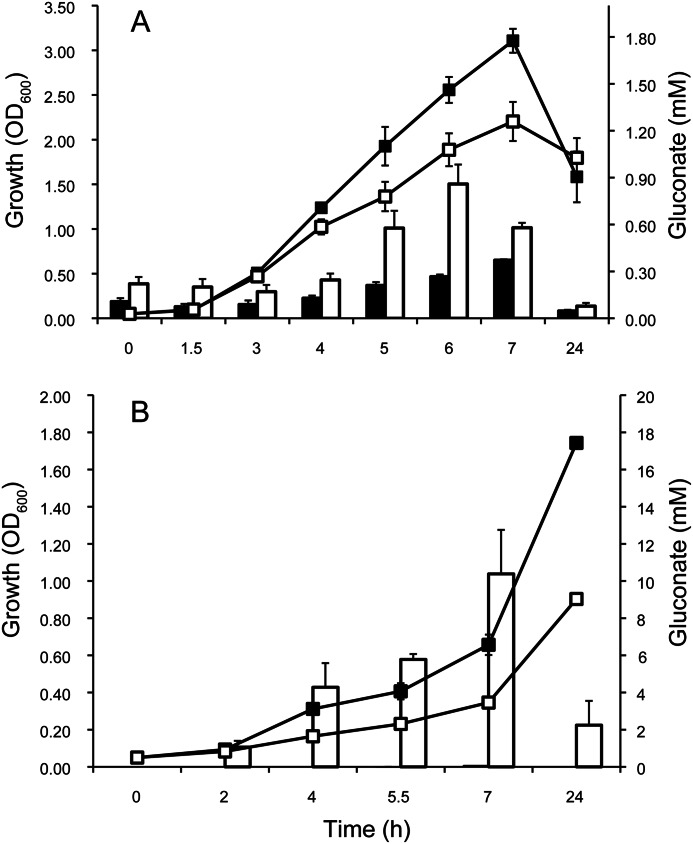
**Extracellular gluconate peaks during growth and declines in stationary phase.** *A*, synthetic cystic fibrosis medium. *B*, minimal medium with glucose as the sole carbon source. *Line plots* represent growth (*left axis*) and *bars* indicate extracellular gluconate production (*right axis*). *Error bars* represent S.E. (*n* = 3). ■, wild type; □, *rpoN* mutant.

Why do RpoN-deficient strains excrete gluconate? The presence of a compound in the culture medium can in general be the result of either the active excretion of the compound or the extracellular conversion of a substrate coupled to the inability to take up the resulting compound quickly enough. Both scenarios can be brought about by the deregulation of metabolic enzymes or metabolite transporters. To narrow down the reason for gluconate production by the *rpoN* mutant, we looked at the kinetics of gluconate production in shake flask culture. Gluconate production peaked at around 6–7 h in both SCFM as well as minimal glucose medium, with a much higher concentration on the glucose medium (Fig. 5). This could potentially be due to gluconate reuptake, but the NMR spectra of supernatants of both cultures showed the production of 2-ketogluconate at similar signal intensities (data not shown). Therefore, a major part of the decrease in gluconate concentration in stationary phase was not due to direct uptake by the cell but to conversion to 2-ketogluconate. Glucose is oxidized to gluconate in the periplasm by a membrane-bound glucose dehydrogenase (GDH), and further oxidation to 2-ketogluconate is carried out by a membrane-bound gluconate dehydrogenase (38). To elucidate whether the increase in gluconate and 2-ketogluconate was caused by decreased uptake or by deregulation of the metabolic enzymes, we measured the activity of both GDH and gluconate dehydrogenase. Gluconate dehydrogenase was not altered, but GDH was clearly deregulated, with more than 5-fold higher activity for the *rpoN* mutant compared with the WT (Fig. 3).

**FIGURE 6.**
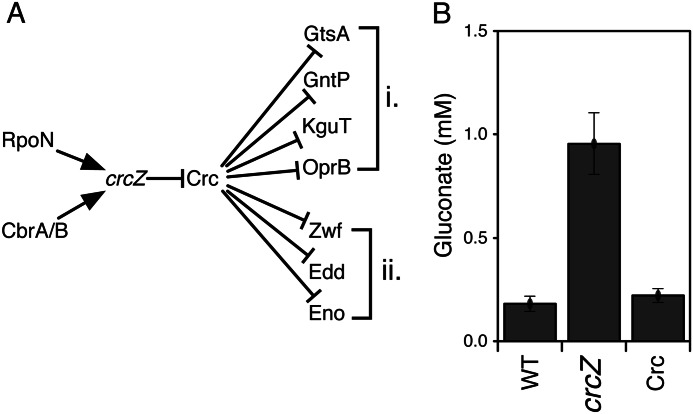
**Gluconate production is regulated by the noncoding small RNA *crcZ*.** *A*, schematic regulatory network of Crc in *P. aeruginosa*. Relevant Crc targets are shown, but this does not represent all possible targets: transporters/permeases (*i*) and metabolic enzymes (*ii*). Gluconate is increased for *rpoN* and *cbrA/B* mutants, so is predicted to be increased for *crcZ* and unaffected by *crc* mutation. *B*, gluconate levels in mid-exponential phase for PAO1 wild-type, a *crcZ* mutant, and a *crc* mutant strain. Gluconate levels were quantified by enzymatic assay (data are mean ± S.E., *n* = 3) and were significantly different (*p* < 0.05) between the WT and *crcZ* deletion strain (Welch's *t* test).

This does not, however, fully explain the gluconate accumulation, as GDH does not have an RpoN binding site^4^. Gluconate was also increased (although to a lesser degree than for *rpoN*) in the *cbrA* and *cbrB* knockout strains (Fig. 2). Both RpoN and CbrA/CbrB have been reported as positive regulators of the non-coding small regulatory RNA *crcZ.* This binds to the catabolite repression protein Crc which, in turn, regulates specific metabolic genes by binding to their mRNA transcripts, reducing translation (39). Hence, we predicted that the *crcZ* knockout strain should also show an increase in gluconate production but the *crc* knockout should not. We obtained both of the knockouts in a PAO1 background and tested them using the gluconate assay. This confirmed our hypothesis. The *crcZ* knockout strain produced significantly more levels of gluconate than the wild type, but the *crc* knockout was not different from the wild type (Fig. 6).

We then asked whether a specific Crc target mediated this effect. Glucose, gluconate, and 2-ketogluconate can be imported through the cytoplasmic membrane by dedicated transporters where they are then phosphorylated by glucokinase, gluconokinase, or 2-ketogluconokinase, respectively. Glucose 6-phosphate and 2-ketogluconate 6-phosphate are further converted to 6-phosphogluconate, which funnels the carbon into the Entner-Douderoff pathway. Several of these genes have Crc binding sites, and so we investigated gluconate production for these mutants (and other adjacent enzymes) (23 additional strains corresponding to 18 additional genes, supplemental Table S4). Only one of these, the *edd* mutant (6-phosphogluconate dehydratase (6PGDH)) produced extracellular gluconate levels comparable with the *rpoN* mutant (Fig. 4). The *edd* and *rpoN* gluconate concentrations were not significantly different (*p* = 0.17).

##### Clinical Relevance

**FIGURE 7.**
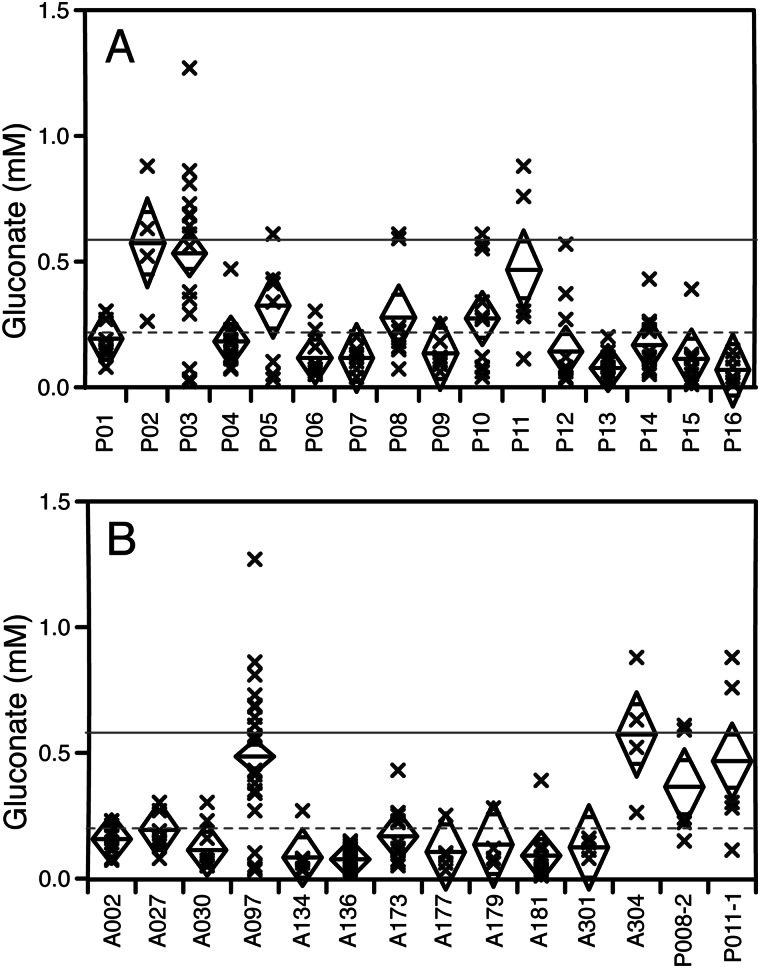
**Clinical isolates from different cystic fibrosis patients produce different amounts of gluconate.** The strains were grown for 5 h, and gluconate was quantified by enzymatic assay. Each point represents the mean gluconate level for an individual strain (*n* = 3). The *center line* of the *diamond* represents the patient mean, and the *vertices* of the *diamond* represent mean ± 95% confidence intervals. The *solid gray line* indicates gluconate production for *rpoN*-mutant PA14, and the *dashed gray line* indicates gluconate production for wild-type PA14. *A*, gluconate production by different strains from 16 individual patients (P01-P16). *B*, gluconate production by different RAPD types (only those types with a minimum of four strains are shown).

Finally, we investigated whether gluconate production from glucose might be of clinical relevance. The CF lung is a nutritionally rich environment, and deregulation of metabolic enzymes and *rpoN* mutations have been reported for populations of clinical isolates (40–45). We profiled gluconate production in a panel of 156 clinical isolates, normalized to optical density. Twenty-five of these isolates had significantly elevated gluconate production after 5 h of growth in SCFM compared with the PAO1 and PA14 wild types (Fig. 7). There was no evidence that gluconate production increased with length of infection (data not shown), but the phenotype was clearly patient-specific, with sets of isolates (*i.e.* clonal lineages) from certain patients expressing high levels of gluconate (*p* < 0.001, Kruskal-Wallis test). Gluconate levels were also significantly related to genotype, as measured by RAPD typing (*p* < 0.001, Kruskal-Wallis test, Fig. 7).

We then went on to measure antibiotic susceptibility in a subset of 96 of these isolates (chosen so that there were six isolates/patient on average) against five antibiotics. Four of the five antibiotics showed clear significant differences between patients and significant associations with the length of infection (data not shown). The fifth, colistin, did not show any significant differences and was excluded from further analyses. We then tested whether susceptibility was related to gluconate levels using a linear model with log_10_(minimal inhibitory concentration) as the dependent variable and “gluconate” and “antibiotic” as continuous and nominal independent variables, respectively. The overall model was weak (R^2^_adj_ = 0.26) but significant. Unsurprisingly, the choice of antibiotic had a highly significant effect (*p* < 0.0001, F = 43.0). Gluconate production was also significant (*p* = 0.0031, F = 8.9). (The interaction term was not significant and was excluded from the model.) The relationship with gluconate was positive so that high gluconate producers tended to have higher log_10_(minimal inhibitory concentration), *i.e.* had reduced susceptibility to antibiotics.

## DISCUSSION

### 

#### 

##### Validating Metabolic Phenotypes

How can one validate the results of this type of project without actually profiling the entire library? There are two basic approaches: to interpret the biochemical changes seen for individual knockouts and to ask whether functionally related genes do, in fact, cluster together (the “FANCY” approach, or functional analysis by correlated responses (4, 6, 46)). We employed both of these methods. There was one obvious case that fulfilled both criteria. The *aceE* and *aceF* genes encode subunits of the pyruvate dehydrogenase complex, clustered together in multivariate analyses, and both mutants had very high concentrations of pyruvate and 2,2′-dihydropropanoate (produced abiotically from pyruvate (47)) in the supernatant. (The 2,2′-dihydropropanoate peak was not fitted but is detectable by simple inspection of the *aceE* and *aceF* spectra.) However, *acoB*, *acoC*, PA14_19900, and *pdhB* are also annotated as putative pyruvate dehydrogenase subunits for *P. aeruginosa* PA14 (48). None of these mutants exhibited a strong pyruvate excretion phenotype, suggesting that these alternative pyruvate dehydrogenases are not actually essential to pyruvate dehydrogenase activity either because they are misassigned for function or were not expressed for *P. aeruginosa* grown in SCFM.

In addition, the two-component system mutants clustered kinase and regulator pairs together where any phenotype was seen, suggesting this could be a relatively simple way to assign “orphan” kinases. This also demonstrates a drawback of this kind of approach. Three of the five pairs did not show any particular phenotype, presumably because the genes were not expressed under the growth conditions. Indeed, for all mutants we looked at, the most distinctive metabolic phenotypes were observed for strains that also had marked growth defects (*aceE*, *aceF*, *rpoN*). This highlights an important point. There is never just one metabolic phenotype of a mutant, as phenotypes are always context-dependent. Extending the footprinting approach to the whole library would, therefore, probably give strong metabolic phenotypes for a relatively small percentage of strains if using just one growth medium. This brings up a second important point. The phenotypes we saw for the growth-restricted mutants were qualitatively different (appearance of unexpected metabolites in the medium) and not just a function of lack of utilization of the original metabolites present. However, this overall extent-of-growth phenotype was the main source of variation in the overall dataset on the basis of the highly significant correlation of PC1 with *A*_600_. Is this kind of metabolic phenotype biologically meaningful, or is it just a trivial observation? Clearly it is meaningful in the basic sense that it encodes information on growth, but it is likely that it may also potentially lead to mistaken hypotheses about functional relatedness solely on the basis of shared growth patterns. Using principal component analysis (or related multivariate approaches) for dimension reduction, therefore, offers the added benefit that this growth effect can be filtered out and the results compared.

A so far unexplained observation was reduced efficiency of lactate utilization by quorum sensing mutants, particularly as this was true for both acyl homoserine lactone and *Pseudomonas* quinolone signal systems and included negative regulators as well as biosynthetic enzymes. This clearly requires further investigation before any conclusions can be drawn, but is of interest given the high concentrations of lactate in CF sputum.

##### Gluconate Excretion by rpoN Mutant Strains

RpoN is an alternative σ factor (σ^54^) that has roles in regulating flagellar synthesis, motility, nitrogen transport, quorum sensing regulation, and virulence, among others (49). Even though RpoN function has been investigated extensively, gluconate production has not, to our knowledge, been identified previously as a phenotype for *rpoN* deletion. We confirmed that the phenotype was neither an artifact of the transposon insertion nor specific to the PA14 background by acquiring a clean in-frame PAO1 deletion mutant. In both backgrounds and in two different media, lack of *rpoN* led to a reduction in growth rate and production of gluconate, which was then further converted to 2-ketogluconate.

Several metabolic phenotypes have already been described for *rpoN* mutants, including the loss of ability to utilize various carbon and nitrogen sources and amino acids (50, 51). RpoN is also important for maintaining the C/N balance in *Pseudomonas* (52–54). Our data are broadly, although not completely, in agreement with these findings. The mutant showed reduced activity on most of the nitrogen sources tested and also failed to grow on succinate, which is expected, because the succinate transporter contains a RpoN binding motif. Interestingly, we observed increased utilization for several carbon sources in the Biolog plates. Because these have only been tested in the microwell plate format using a redox dye, it remains to be seen whether these observations would hold true for growth under standard shake flask conditions.

The metabolomic characterization of the *rpoN* mutant found several additional phenotypes that occurred across different metabolic pathways and were at least partly growth phase-dependent. Citrate and 4-hydroxyphenylacetate were the only metabolites that had consistent RpoN-dependent changes in both exponential and stationary phase growth (5 h and 24 h). The lower levels of citrate could be the result of increased isocitrate lyase activity, which was shown to be elevated in an *rpoN* mutant of *P. aeruginosa* (41). The higher level of 4-hydroxyphenylacetate is probably linked to altered regulation of aromatic amino acid metabolism. Previous studies have reported that an aromatic amino acid transporter is also a Crc target (55).

Because of the pleiotropic effects of RpoN and the complex regulation of central metabolism, it is not possible to provide simple explanations (“just-so stories”) for all observed metabolic changes. However, some of them can be attributed to altered catabolite repression. Catabolite repression in *P. aeruginosa* is different from the well studied Crp-mediated system of enterobacteria. In *E. coli*, catabolite repression mainly regulates sugar uptake, with glucose being the preferred substrate, and is governed by the phosphorylation state of the phosphotransferase system transporters (39, 56). In contrast, catabolite repression in *Pseudomonas* extends to amino and organic acids (39), with data for *P. aeruginosa* and *Pseudomonas putida* clearly showing a distinct hierarchy of metabolite uptake from complex growth media (57, 58). Furthermore, although glucose is the preferred substrate in enterobacteria, pseudomonads preferentially take up citric acid cycle intermediates, such as succinate, and certain amino acids, reflected by the poor correlation between glucose uptake and growth shown in this study. The Crc protein is a posttranscriptional inhibitor that controls the expression of over 100 genes by binding to mRNA and preventing translation (58, 59). The active levels of Crc are regulated by CrcZ, a non-coding RNA that is under positive transcriptional control by RpoN (60). A mutation in *rpoN* would therefore result in lower CrcZ levels which, in turn, would lead to deregulation of the proteins normally controlled by Crc.

Several of the phenotypes observed here could be due to direct involvement of the Crc response. Proteins predicted to be targets of Crc include the proline/sodium symporter (*putP*), the lactate permease (*lldP*), and an alanine/sodium symporter (56), and proline, lactate, and alanine levels were higher in *rpoN* supernatants. Another target is *phhA* (phenylalanine-4-hydrolase), which converts phenylalanine to tyrosine, and intracellular aromatic amino acid concentrations were affected in the mutant, indicating a deregulation of amino acid metabolism.

However, was the gluconate excretion phenotype regulated via *crcZ*? A strong hint was that gluconate was also increased for the *cbrA/cbrB* mutants, which, like RpoN, are positive regulators of *crcZ*. We then confirmed this by analyzing *crcZ* and *crc* knockouts. However, this still does not identify a specific target, as there are several relevant genes in *P. aeruginosa* with Crc-binding motifs, including both metabolite transporters and metabolic enzymes (56). To follow this up, we screened an additional set of Crc-regulated mutants and found that 6PGDH reproduced the RpoN phenotype. This has a direct mechanistic explanation. Metabolites upstream from 6PGDH accumulate in the cell, and gluconate is exported to relieve this build-up. In addition, glucose dehydrogenase activity is increased in the *rpoN* mutant, which would tend to increase gluconate buildup as well. However, we do not as yet have a direct explanation for this observation. It is still possible that the regulation by Crc could be more distributed. Perhaps knockdowns of multiple transporters, for instance, would contribute to the extracellular gluconate build-up. However, given that the 6PGDH gluconate excretion phenotype is not significantly different from the *rpoN* mutant, it is clearly more parsimonious to conclude that the effect is modulated via 6PGDH.

##### Clinical Relevance

It is known that *P. aeruginosa rpoN* mutates sporadically in the CF lung, and this represents a potential adaptation (45). Several clinical isolates were gluconate producers when grown in SCFM, with significant differences between strains isolated from different patients. Further studies could be carried out to investigate whether the isolates are indeed *rpoN* mutants and whether *rpoN* mutations are always linked to gluconate production or whether they are compensated by secondary suppressor mutations, as shown for *mucA* and alginate production (61, 62).

Although no obvious selective advantage was detected, *i.e.* gluconate production was not associated with length of infection, there were differences between genotypes. Four of the RAPD types were high gluconate producers, including the A097 type that was seen in multiple patients from the collecting laboratory (29). However, the A002 type that was also found in multiple patients was a low gluconate producer, so there is no apparent relationship between potential transmissibility and gluconate production.

We measured susceptibility of a subset of the clinical isolates to four antibiotics used in treating chronic *P. aeruginosa* CF infections (63). This was significantly, although weakly, related to gluconate production. Rapid forms of atmospheric pressure mass spectrometry could potentially be used to assign bacterial strains directly from patient samples such as sputum, without culturing, for clinical diagnostics (64). It is easy to see how this could be extended to measure bacterially produced metabolites in biofluids. Hence, although we do not claim that gluconate production would be directly useful in a clinical setting, it serves as proof of principle for how observations from untargeted profiling experiments can ultimately be linked to clinically relevant phenotypes. In general, context is increasingly seen as important for antibiotic susceptibility of microbes with a realization that there is no one Platonic value for resistance or sensitivity for a particular strain (65). Understanding the interactions of the metabolic network with antibiotic resistance is an important future goal (66).

## Supplementary Material

Supplemental Data

## Footnotes

4 D. Studholme, personal communication.

## Footnotes

3 The abbreviations used are:

CF: cystic fibrosis
SCFM: synthetic cystic fibrosis medium
GDH: glucose dehydrogenase
6PGDH: 6-phosphogluconate dehydratase.
