# Supplementary material for: Metabolite Profiling to Characterize Disease-related Bacteria: GLUCONATE EXCRETION BY PSEUDOMONAS AERUGINOSA MUTANTS AND CLINICAL ISOLATES FROM CYSTIC FIBROSIS PATIENTS
Source: J Biol Chem. 2013 Apr 9;288(21):15098–109. doi: 10.1074/jbc.M112.442814 (PMC3663530; doi:10.1074/jbc.M112.442814)
Supplement: Supplemental Data [file supp_288_21_15098__index.html]

Metabolite profiling to characterize disease-related bacteria: gluconate excretion by Pseudomonas aeruginosa mutants and clinical isolates from cystic fibrosis patients — Metabolite Profiling to Characterize Disease-related Bacteria — P. aeruginosa Metabolic Footprinting — Supplemental Data 

# Metabolite Profiling to Characterize Disease-related Bacteria

## Supplemental Data

**Files in this Data Supplement:**

- Supplemental Table S1 (.xlsx, 37 KB) - List of initial set of mutants analysed in the study
- Supplemental Table S2 (.xlsx, 109 KB) - Fitted metabolite concentration data
- Supplemental Table S3 (.xlsx, 46 KB) - Biolog Phenotype Microarray results for PA14 wild-type and rpoN mutant
- Supplemental Table S4 (.xlsx, 34 KB) - List of additional mutants analysed in the study
